# The treatment of challenging visceral and peripheral arterial aneurysms with flow diversion—a German multicentric observational study

**DOI:** 10.3389/fmedt.2025.1693030

**Published:** 2026-01-06

**Authors:** Stefan Schob, Jörg Ukkat, Gregor Scharf, Yvonne Maybaum, Lea Tauber, Clara Lüdeke, Endres John, Karl Julius Büchner, Robert Fiedler, Christian Frahm, Mathias Wieprecht, Silvio Brandt, Mohamed Alsabbagh, Ararat Minasyan, Andreas Simgen, Richard Brill, Jakob Albrecht, Walter Alexander Wohlgemuth, Maximilian de Bucourt, Marie-Sophie Schüngel

**Affiliations:** 1 Univsersitäts- und Poliklinik für Radiologie, Universitätsklinikum Halle, Halle, Germany; 2 Universitäts- und Poliklinik für Viszerale, Gefäß- und Endokrine Chirurgie, Universitätsmedizin Halle/Saale, Halle (Saale), Germany; 3 Universitätsklinikum Regensburg, Interdisziplinäres Zentrum für Pädiatrisch-interventionelle Radiologie, Regensburg, Germany; 4 Clinic for Neuroradiology, St. Georg Hospital, Leipzig, Germany; 5 Evangelisches Krankenhaus Königin Elisabeth Herzberge, Innere Medizin I, Berlin, Germany; 6 Klinik für Diagnostische und Interventionelle Radiologie, Harzklinikum Dorothea Christiane Erxleben, Wernigerode, Germany; 7 Radiologisches Institut, Alexianer St. Josephs Krankenhaus Potsdam, Potsdam, Germany; 8 Institut für Radiologie, Asklepios Klinikum Weißenfels, Weißenfels, Germany; 9 Institut für Radiologie und Neuroradiologie, Klinikum Chemnitz, Chemnitz, Germany; 10 Klinik für Neuroradiologie, Westpfalz Klinikum Kaiserslautern, Kaiserslautern, Germany; 11 Klinik für Radiologie, Charité – Universitätsmedizin Berlin, Berlin, Germany

**Keywords:** flow diversion, visceral and peripheral arterial territories, tortuous parent vessels, aneurysms involving a bifurcation, flow diverter stents, antiplatelet therapy, platelet function, stent occlusion

## Abstract

**Purpose:**

Endovascular treatment of visceral and peripheral aneurysms has become the modality of choice over the past few decades. A variety of different techniques are being applied, including deconstructive approaches, such as parent vessel occlusion with coils or liquid embolic agents, and reconstructive approaches, such as stent grafting, stent-assisted coiling, or balloon-assisted coiling. Reconstruction is preferred over vessel or segmental sacrifice due to the risk of ischemia in the dependent territory. However, conventional reconstructive techniques fail, possibly due to the tortuosity of the parent artery or involvement of arterial bifurcations. Flow diversion, a comparatively novel method using densely braided stents, has become well established as a therapeutic option for the treatment of uncoilable or failed aneurysms in cerebral circulation because flow diverter stents can be implanted in highly tortuous vessels and can also be used to treat bifurcation aneurysms without causing occlusion of non-collateralized side branches under sufficient inhibition of platelet function. A few reports have been published on the use of flow diverter stents for the treatment of aneurysms in visceral or peripheral circulation, but systematic studies are lacking. The purpose of this multicenter study is to summarize the experience of different vascular centers that have utilized flow diversion techniques in peripheral vasculature.

**Materials and methods:**

This multicentric retrospective analysis includes data from 10 vascular centers on treatments performed between 2022 and 2025. Aside from the safety and feasibility of the approach, procedural aspects, antiplatelet medication, follow-up results, and complications were investigated in a retrospective manner.

**Results:**

A total of 36 patients (22 female, with a mean age of 59.2 years, ranging between 36 and 80 years), one with two aneurysms, were treated in the following arterial territories with decreasing frequency: splenic artery (*n* = 18), hepatic arteries (*n* = 8), renal artery (*n* = 7), superior mesenteric artery (*n* = 1), gastroduodenal artery (*n* = 1), external iliac artery (*n* = 1), and inferior genicular artery (*n* = 1). From a technical perspective, all treatments were performed successfully. A total of four aneurysms (11%) were occluded immediately at the end of the procedure, while in 33 aneurysms (89%), the perfusion was reduced to a varying extent. At the first follow-up study scheduled after 3 months, 17 aneurysms (46%) were occluded, 14 (38%) showed reduced perfusion, and the remaining six patients had not yet undergone follow-up. At 12 months following the procedure, 26 patients with lesions underwent follow-up. A total of 27 aneurysms (73%) were occluded after 12 months, with 17 of them already examined at the 3-month study, two of those without a follow-up at 3 months and one exhibiting a small neck remnant. At this point, 11 patients with aneurysms had not yet attended the second follow-up study. Complications occurred in two patients (5%); one was related to clopidogrel resistance and manifested as stent occlusion during the intervention, while the other was related to paused antiplatelet therapy due to hemorrhage unrelated to the treated aneurysm and manifested with stent occlusion during the early postinterventional period. Both patients recovered well. Antiplatelet medication differed substantially between the centers.

**Conclusion:**

Our multicenter observational study demonstrated the excellent safety and efficacy of flow diversion as an alternative treatment modality for visceral and peripheral aneurysms. The complication rates were low, and all complications were related to insufficient inhibition of platelet function during the intervention or in the early postinterventional phase, which can be addressed by platelet function testing prior to the interventions, followed by tailored antiplatelet therapy.

## Highlights

- Flow diversion seems to be a good therapeutic option for challenging aneurysms of the visceral and peripheral arterial territories; tortuous parent vessels and aneurysms involving a bifurcation are particularly suitable for flow diverter stents.
- Occlusion of covered arteries did not occur under suitable antiplatelet therapy during short-term observation.
- Although rare, high on-treatment platelet function can result in early stent occlusion with ischemic sequelae; thus, platelet function testing and tailored antiplatelet therapy should be utilized.

## Introduction

Flow diversion (FD) has significantly improved the endovascular armamentarium for the treatment of challenging cerebral aneurysms and has now been routinely used in clinical practice for nearly 25 years. The therapeutic goal of FD is the reconstruction of the vessel wall along the aneurysmatic vessel segment without using foreign material within the aneurysm sac. Flow diverter stents (FDS) are made of a densely braided mesh composed of either nitinol or cobalt–chromium wires in varying combinations with platinum. A flow diverter mesh achieves a metal surface coverage ranging from 22% to 70%, which is significantly higher than the metal surface coverage achieved by conventional self-expanding stents (1). The dense mesh of an FDS covering an aneurysm causes two main events. At first, hemodynamic downgrading manifests immediately after the implantation, as the device strongly reduces aneurysm perfusion, lowers wall shear stress, disrupts vortices, induces flow stagnation, and redirects blood flow away from the sac. Fibrin accumulation at the surface of the FDS further contributes to these hemodynamic effects (3). The decreased perfusion results in progressive laminar thrombosis within the aneurysm (2). After a few weeks, a neointima forms along the mesh of the FDS, which mainly consists of smooth muscle cells and endothelium. This process does not depend on the formation of a clot inside the aneurysm, but is linked to the ingrowth of smooth muscle and endothelial cells from the proximal and distal landing zones of the implant (4), which must therefore achieve a good circumferential adherence to the healthy vessel wall proximal and distal to the aneurysm (2). If a complete neointima has formed along the scaffold of the FDS, the aneurysm shrinks and contains organized connective tissue; however, if incompletely occluded aneurysms are formed, they are characterized by large spaces without neointima at the scaffold surface and differing amounts of organized connective tissue together with fresh thrombus material (2).

Overall, therapeutic success increases over time and is achieved in the vast majority of cases with complete occlusion in more than 75% of cases after 1 year and more than 95% of cases after 5 years (5) in the cerebral vasculature.

Following the success of FD in the treatment of intracranial aneurysm(6–9), the technique has also been recognized as a promising option for visceral and renal artery aneurysms (10–13). What makes this technique valuable in the treatment of the complex vascular visceral and renal anatomy—in particular if bifurcations are involved—is the fact that covered side branches without sufficient collateralization can remain patent, while the aneurysm is occluded with time (9). However, clinical evidence in this regard is lacking, with the Society for Vascular Surgery and the European Society for Vascular Surgery considering FD as a promising endovascular technique, yet one that has not been completely elucidated.

As a consequence, our study aims to provide further evidence for the safety and feasibility of FD for the treatment of visceral, renal, pelvic and other peripheral arterial aneurysms, putting special focus on antiplatelet therapy, technical considerations, technical and clinical complications and follow up results.

## Materials and methods

This multicentric observational study includes patient data from 10 centers for treatments performed between 2022 and 2025. Patients were treated using the Derivo peripher (Acandis, Pforzheim, Germany), the Derivo2Heal—which has an antithrombogenic coating consisting of covalently bound fibrin and heparin—or the Pipeline Flex Vantage, which features another antithrombogenic coating (Shield technology) consisting of phosphorylcholine, which mimics the surface of red blood cells (Medtronic, Irvine, California).

Anonymized information, including patient charts, antiplatelet therapy protocol, imaging studies, and procedural details, including all available follow-up data, was retrospectively reviewed.

Our institutional ethics board approved the study (number 2025-115) and waived the need for informed consent due to its retrospective nature.

In terms of the outcome, the primary item analyzed in our study was aneurysm occlusion. The other investigated items were individual antiplatelet therapy and its duration; ischemic and hemorrhagic complications associated with the procedure and the implanted devices; device complications such as FDS migration, fishmouthing, and thrombotic occlusion; and the need for further implants. Platelet function testing was not routinely conducted in all centers; therefore, platelet reactivity under antiplatelet therapy was evaluated only if available. Table 1 provides an explanatory overview of the study endpoints. Table 2 summarizes the details of all included patients.

**Table 1:** Overview of the study endpoints.

Endpoints	Definition	Evaluation method	Comment
Aneurysm occlusion (primary endpoint)	Extent of aneurysm occlusion, related to intraaneurysmal thrombosis and formation of a neo-intima along the aneurysm neck	Immediately after the procedure with digital subtraction angiography, in the follow-up studies, DSA, computed tomography angiography, or magnetic resonance angiography using the ÒKelly-Marotta aneurysm occlusion scale	Aneurysm occlusion with flow diversion occurs over time and is related to thrombus formation within the aneurysm, largely depending on the hemodynamic flow-diverting effect of the implants and, more importantly, the ingrowth of a continuous layer of neo-intima along the struts of the implant
Parent vessel patency (secondary endpoint)	Physiological perfusion of the vessel segment carrying the flow diverter stent construct	Angiographic follow-up study, consisting of catheter angiography, computed tomography angiography, or magnetic resonance imaging angiography	Flow diverter stents strongly alter the flow profile in a treated vessel segment and, especially in the case of insufficient antiplatelet therapy, may cause unintended parent vessel occlusion
Technical success (secondary endpoint)	Procedurally uneventful implantation of the flow diverter construct, including maneuverability of the microcatheters distal to the aneurysm, sufficient flow diverter stent opening, and achievement of sufficient proximal and distal landing zones in the parent artery	Fluoroscopy and digital subtraction angiography. Fluoroscopic imaging is used to assess the implant during deployment and after implantation. Catheter angiography is used to assess the perfusion of the parent artery territory after stenting and the hemodynamic effect of the flow diverter construct on aneurysm perfusion	Overcoming substantial tortuosity of arteries with aneurysms is one of the key features of flow-diverting interventions. Therefore, the required flexibility of the catheter and stent materials comes at a higher risk for technical complications, such as stent twisting, non-opening, and implant dislocation during and after the procedure, carrying the risk of subsequent parent vessel occlusion
Need for additional implants to achieve treatment success (secondary endpoint)	Required use of additional implants, such as balloon-mounted stents and self-expanding laser-cut stents to create a sufficiently working flow diverter stent construct	Imaging of the implants and the treated parent vessel segment, as explained previously	In some cases, implants with greater radial force, such as balloon-mounted stents or self-expanding laser-cut stents, are necessary to optimize the opening behavior or anchoring of flow-diverting stents. For example, if a flow diverter stent ends in a very curved segment, its opening can be impaired. This can be successfully managed with the aforementioned implants
High on-treatment platelet function (antiplatelet drug resistance, secondary endpoint)	All of the currently used antiplatelet drugs, despite being administered accordingly, may not cause sufficient platelet function inhibition in individual patients. This phenomenon is termed “high on-treatment platelet function.” Most frequently this phenomenon occurs with clopidogrel and is also known as “clopidogrel resistance”	Different platelet function testing systems are available, for example, the Multiplate platelet function analyzer, the Verify Now system, or the Platelet Function Analyzer (PFA-200). Platelet function testing is not routinely required for stenting procedures in the guidelines; therefore, not all centers use it. When employed, the Multiplate system was used in our study	Platelet function testing can evaluate the extent of platelet function inhibition of different antiplatelet drugs, such as acetylic salicylic acid, clopidogrel, prasugrel, and ticagrelor. In our opinion, platelet function testing should be mandatory to achieve satisfactory safety profile of stenting procedures, especially when using flow diverter stents
Implant insufficiency (secondary endpoint)	Implant insufficiency includes stent deformities, for example, fishmouthing and inappropriate apposition to the vessel wall	Implant insufficiency is best evaluated by fluoroscopy, catheter angiography, or computed tomography angiography, all being modalities that are able to show the implants in relation to the treated vessel segment	Implant insufficiencies most frequently occur during or at the end of the implantation. In a few cases, those stent deformities may develop in the follow-up period; in our patient collective, only proximal fishmouthing occurred
Complications (secondary endpoint)	Significant complications are either hemorrhagic or ischemic in nature and can manifest in the parent vessel, at the site of the vascular access, or remotely, related to the platelet function inhibition	The available imaging data, patient interviews after the intervention and the follow-up, and physical examination after the procedure and during the follow-ups were reviewed	In general, access site complications, such as femoral or retroperitoneal hematomas, are comparatively frequent, but did not occur in our patients. There were no hemorrhagic complications, but two ischemic complications related to high on-treatment platelet function

**Table 2:** Summary of the treatment aspects of all patients.

Patient number	Sex	Age	Parent artery	Aneurysm features	Flow diverter, stents	Postinterventional outcome	3 months FU	12 months FU	First antiplatelet	Second antiplatelet	Fish-mouthing requiring stenting	FDS-occlusion	High on treatment platelet reactivity
1	Male	60	Renal	Saccular, unruptured 25 mm	Derivo peripher 7 × 50 mm *N* = 2	OKM A2	OKM B2	OKM D	ASA	Prasugrel	No	Yes, during FU abdominal hemorrhage, antiplatelet therapy stopped	No
2	Male	69	Renal	Saccular, unruptured 30 mm	Derivo peripher 5.5 × 30 mm 5.5 × 30 mm *N* = 2	OKM A2	OKM B2	OKM C3	ASA	Clopidogrel	No	No	No
3	Male	46	Splenic	Saccular, acutely ruptured 8 mm	Pipeline Flex Vantage 2.75 × 20 mm *N* = 1	OKM C3	OKM D	OKM D	ASA	Clopidogrel	No	No	No
4	Male	42	Genicular, left lateral inferior	Saccular, unruptured 23 mm	Derivo peripher 6 × 40 mm, Derivo2Heal 5 × 15 mm *N* = 2	OKM A3	OKM D	OKM D	ASA	Clopidogrel	No	No	No
5	Female	46	Splenic	Saccular, unruptured but with significant growth within 6 months 20 mm	Derivo peripher 4.5 × 25 mm 4.5 × 30 mm 4.5 × 40 mm *N* = 3	OKM A3	OKM D	OKM D	ASA	Clopidogrel	No	No	No
6	Female	75	Splenic	Saccular, unruptured 31 mm	Derivo peripher 6 × 40 mm *N* = 2	OKM A3	OKM D	OKM D	ASA	Clopidogrel	Yes Acclino Flex Plus Heal 5.0 × 20 mm	No	No
7	Female	54	Renal	Fusiform and saccular, known fibromuscular dysplasia 21 mm	Derivo peripher 7 × 40 mm *N* = 1	OKM A3	OKM D	OKM D	ASA	Prasugrel	No	No	No
8	Male	69	Hepatic	Saccular, rapid growth over 6 months 28 mm	Derivo peripher 8 × 30 mm *N* = 1	OKM B3	OKM D	OKM D	ASA	Clopidogrel	No	No	No
9	Female	54	Splenic	Saccular, unruptured 28 mm	Derivo peripher 8 × 50 mm 7 × 40 mm *N* = 2	OKM D	OKM D	OKM D	ASA	Clopidogrel	No	Yes	Yes
9	Female	54	Superior mesenteric	Saccular, unruptured but rapid growth over 6 months 18 mm	Derivo peripher 7 × 30 mm *N* = 1	OKM D	OKM D	OKM D	ASA	Clopidogrel	No	Yes	Yes
10	Male	62	Hepatic	Saccular, ruptured	Derivo2Heal 5.5 × 25 mm 5.5 × 25 mm 5.5 × 30 mm *N* = 3	OKMA3	OKM D	OKM D	Ticagrelor	None	No	No	No
11	Female	38	Splenic	Saccular, unruptured Rapid growth over 6 months 33 mm	Derivo2Heal 6 × 25 mm 6 × 30 mm 6 × 30 mm 6 × 40 mm *N* = 4	OKM B3	OKM D	OKM D	Prasgurel	None	No	No	No
12	Male	59	Splenic	Saccular, unruptured 22 mm Polycystic kidney disease	Derivo peripher 6 × 30 mm *N* = 1	OKM A3	OKM B3	OKM D	ASA	Prasugrel	No	No	No
13	Male	48	Hepatic	Saccular, ruptured 20 mm	Derivo2Heal 4 × 15 mm 4 × 15 mm 4 × 20 mm 4 × 20 mm 4 × 25 mm *N* = 5	OKM C3	OKM D	OKM D	ASA	Ticagrelor	No	No	No
14	Female	67	Renal (l)	Saccular, unruptured 25 mm	Derivo2Heal 6 × 30 mm *N* = 1 Acclino Flex Heal 6.5 × 20 mm	OKM B3	OKM C3	OKM D	ASA	Prasugrel	Yes	No	No
15	Female	72	Splenic	Saccular, unruptured 23 mm	Derivo peripher 6 × 50 mm *N* = 1	OKM A3	OKM C3	OKM D	ASA	Clopidogrel	No	No	No
16	Female	63	Splenic	Saccular, unruptured 28 mm	Derivo peripher 8 × 50 mm *N* = 1	OKM B3	OKM C3	OKM D	ASA	Clopidogrel	No	No	No
17	Male	56	External iliac	Fusiform, unruptured 72 mm	Derivo peripher 8.0 × 50 mm 8.0 × 40 mm 8.0 × 40 mm 8.0 × 30 mm 8.0 × 30 mm 8.0 × 25 mm *N* = 6	OKM C2	OKM C3	OKM D	ASA	Prasugrel	No	No	No
18	Male	61	Renal	Saccular, unruptured 15 mm	Derivo peripher 8 × 50 mm *N* = 1	OKM A3	OKM D	OKM D	ASA	Clopidogrel	No	No	No
19	Male	55	Hepatic	Saccular, ruptured 14 mm	Derivo peripher 7 × 30 mm 6 × 40 mm *N* = 2	OKM D	OKM D	OKM D	ASA	Prasugrel	No	No	No
20	Female	47	Hepatic	Saccular, ruptured 14 mm	Derivo peripher 5.5 × 25 mm 6 × 20 mm *N* = 2	OKM D	OKM D	OKM D	ASA	Prasgurel	No	No	No
21	Female	80	Splenic	Saccular, unruptured 26 mm	Derivo peripher 6 × 15 mm 6 × 20 mm *N* = 2	OKM A2	OKM A3	OKM D	ASA	Clopidogrel	No	No	No
22	Female	76	Splenic	Saccular, multilobulated with daughter aneurysms unruptured 25 mm	Derivo peripher 6 × 30 mm 6 × 25 mm 5.5 × 30 mm *N* = 3	OKM A1	OKM D	OKM D	ASA	Prasugrel	No	No	No
23	Female	59	Splenic	Saccular, multilobulated with daughter aneurysms unruptured 25 mm	Derivo peripher 8 × 40 mm *N* = 1	OKM A1	OKM B 3	OKM D	ASA	Prasugrel	No	No	No
24	Female	80	Splenic	Saccular, multilobulated, involving the mainstem bifurcation 31 mm	Derivo peripher 6 × 30 mm *N* = 1	OKM A3	NA	NA	ASA	Prasugrel	No	No	No
25	Female	56	Renal	Saccular, unruptured 15 mm	Derivo peripher 6 × 20 mm; *N* = 2	OKM A2	NA	NA	ASA 100 mg	Clopidogrel 75 mg	No	No	NA
26	Male	78	Splenic	Saccular, unruptured 87 mm	Derivo peripher, 8 × 50 mm; Derivo peripher, 8 × 25 mm; *N* = 2	OKM A3	OKM B3	NA	ASA 100 mg	Clopidogrel 75 mg	No	No	NA
27	Female	58	Renal	Saccular, unruptured 14 mm	Derivo peripher 5 × 40 mm; *N* = 1	OKM A2	NA	NA	ASA 100 mg	Clopidogrel 75 mg	No	No	NA
28	Female	36	Splenic	Saccular, unruptured 26 mm	Derivo peripher, 6 × 30 mm; *N* = 1.	OKM A3	OKM B3	OKM D	ASA 100 mg	Clopidogrel 75 mg	No	No	NA
29	Male	67	Splenic	Saccular, unruptured 53 mm	Derivo peripher, 8 × 50 mm; *N* = 1 Accero Rex Stent, 10 × 60 mm; *N* = 1	OKM A2	OKM C2	OKM D	ASA 100 mg	Clopidogrel 75 mg	No	No	NA
30	Female	64	Splenic	Saccular, unruptured 22 mm	Derivo peripher, 6 × 40 mm; *N* = 1	OKM A2	NA	NA	ASA 100 mg	Clopidogrel 75 mg	No	No	NA
31	Female		Splenic	Saccular, unruptured 27 mm	Derivo peripher 6 × 30 mm 6 × 50 mm *N* = 2	OKM B2	NA	NA	ASA 100mg	Ticagrelor 180mg	No	No	No
32	Female	58	Splenic	Saccular, unruptured 25 mm	Derivo peripher, 7 × 50 mm, Derivo peripher 7 × 40 mm, *N* = 2 Accero Rex Stent 7 × 30 mm *N* = 1	OKM A3	n/a	n/a	ASA	Clopidogrel	Yes,	No	No
33	Female	70	Common hepatic artery	Fusiform, unruptured 30 mm	Derivo peripher, 8 × 50 mm, *N* = 1 10 × 30 mm Accero Rex Stent *N* = 1	OKM A3	OKM B3	n/a	Edoxaban	ASA	No	No	No
34	Female	41	Aberrant common hepatic artery (arising from SMA)	Saccular, unruptured 40 mm	Derivo peripher, 8 × 40 mm, 8 × 50 mm, 8 × 50 mm, *N* = 3 Cook formula stent 7 × 16 mm	OKM A3	OKM D	n/a	ASA	Clopidogrel	No	No	No
35	Male	54	Common hepatic artery	Saccular, unruptured 20 mm	Derivo peripher, 8 × 50 mm *N* = 1	OKM A3	OKM D	n/a	ASA	Clopidogrel	No	No	No
36	Male	50	Gastroduodenal artery	Saccular, unruptured 25 mm	Derivo peripher, 8 × 40 mm *N* = 1	OKM A3	OKM B2	n/a	ASA	Clopidogrel	Yes (re-intervention after 6 months, 7 × 20 mm cook formula stent)	No	No

### Inclusion and exclusion criteria

All patients who were treated with flow diversion for aneurysms of the visceral arteries—including the splenic, hepatic, mesenteric, gastroduodenal, and renal arteries—as well as for aneurysms of the iliac arteries and branches of the popliteal arteries were included in our study. The indications for treatment were in accordance with the current guideline of the Society for Vascular Surgery. Completion of the postinterventional follow-up studies was not mandatory although strongly recommended, as the primary focus of our investigation was safety and feasibility of flow diversion as a novel approach for peripheral aneurysms.

Patients who were treated with flow diversion for aneurysms of the supra-aortic extracranial arteries were excluded from our study.

### Angiographic approach

In general, vascular access was achieved using the right common femoral artery via the Seldinger technique using an 8 French sheath. A bolus of 5,000 international units of heparin was given after the groin puncture. For catheterization of the target vessel, a diagnostic catheter with vertebral configuration, Simmons 1 configuration, RIM1 configuration, Mikkaelson configuration, or USL2 configuration was used to catheterize the target vessel. Either a long wire exchange maneuver or a larger sheath (8 French) was used to then advance an intermediate catheter (Sofia EX, 105 cm or Sofia 5F 115 cm, both Microvention, Costa Rica) within the parent vessel, providing sufficient stability for the FDS implantation via the microcatheter. In cases of extraordinarily challenging anatomical circumstances, when the required microcatheter could not be navigated to the aneurysmatic vessel, a 0.017 in. microcatheter was used to navigate. Then, a stent retriever anchoring maneuver was performed as described previously (14). This maneuver uses highly maneuverable 0.017 in. microcatheters to navigate distal to the target segment. Then, a suitable stent retriever was placed transiently in this segment, and the smaller microcatheter, which cannot be used to implant the FDS, was removed. Subsequently, the carrier wire of the stent retriever *in situ* was used to advance the needed delivery microcatheter. After atraumatic removal of the stent retriever, the FDS was implanted. According to the preinterventional imaging, predominantly computed tomography angiograms, the FDS was sized considering the diameter of the distal and proximal landing zones and the diameter of the aneurysmatic segment itself. In peripheral FD, subtle oversizing was applied. For example, for a 5-mm target vessel, a 6-mm FDS was used. The implantation was performed under fluoroscopic imaging, using unsubtracted and subtracted digital angiography (DSA) for proper FDS placement. At the discretion of the individual operator, one or more FDSs were implanted (using a telescoping technique). Using overlapping FDS stents, the hemodynamic effect can be increased. If the clinical situation does not allow a prolonged process of aneurysm occlusion, for example, in the context of a ruptured aneurysm or rapid growth within a short period of time, more than one FDS can be used to increase the hemodynamic effect and subsequently shorten the process of complete intraaneurysmal thrombosis significantly. A few cases required the use of additional stents. The details are provided in Table 1.

After the procedure, patients were monitored at an intermediate care unit and transferred to the ward for surveillance at the discretion of the operator.

### Antiplatelet medication

Antiplatelet medication differed between the centers. In most cases, dual antiplatelet therapy was performed using acetylic salicylic acid (ASA) as a COX inhibitor and a P2Y12 inhibitor (clopidogrel, prasugrel, or ticagrelor). Antiplatelet therapy in elective cases was initiated with a loading dose at least 24 h prior to the intervention consisting of 500 mg ASA orally in combination with clopidogrel (300 mg orally), prasugrel (30 mg orally), or ticagrelor (180 mg orally).

In the case of dual antiplatelet therapy, after loading with the respective antiplatelet drug, the medication was continued with daily doses of 100mmg ASA in combination with 75 mg clopidogrel, 10 mg prasugrel, or 180 mg ticagrelor (the last being given in two doses of 90 mg 12 h apart). Dual antiplatelet therapy was performed for 3 months and then changed to ASA monotherapy lifelong. Mono antiplatelet therapy was performed in a few cases for which the details are given in the following.

### Evaluation of aneurysm occlusion

At the end of the procedure, the hemodynamic effect of the implanted FDS was assessed using the ÒKelly-Marotta scale (15). In brief, the volume of the residually perfused aneurysm and the duration of contrast agent stasis relative to the time point of contrast washout were assessed. If the aneurysm remained completely perfused, it was graded as OKM A; if it was only partially perfused, but there was more than a neck remnant, it was graded as OKM B; if there was only a neck remnant, it equaled OKM C; and if it was completely occluded, it was considered OKM D. With regard to the length of the contrast stagnation, 1 was assigned if the contrast agent was cleared during the arterial phase, 2 if the contrast agent was washed out after the arterial but before the venous phase, and 3 if the contrast agent remained within the aneurysm sac as long as the venous structures were opacified in the DSA run.

The first follow-up imaging was scheduled 3 months after the procedure and the second mandatory follow-up imaging was scheduled 12 months following the procedure. Some centers performed additional follow-ups earlier, for example at 6 months and at 9 months following the procedure. Patient data for those who had not yet undergone postinterventional follow-up, due to the recency of their treatment, were also reviewed. Pending follow-up investigations were labelled as not available (n.a.). DSA, CT angiography, or MR angiography was performed as the imaging modality for the follow-up studies. In follow-up imaging, aneurysm occlusion, stent patency and configuration, and opacification of FDS-covered side branches were assessed. The dependent organ was screened for potential ischemic defects.

## Results

### Procedural details, implants, and microcatheter setup

The anesthesia utilized for the procedures differed between the centers. A total of 16 treatments were performed under local anesthesia and 19 treatments were performed under general anesthesia.

In the majority of cases, a Derivo peripher was used. This is an FDS specifically indicated for use in abdominal and renal arteries, composed of a nickel–titanium alloy (nitinol). The device is a second-generation flow diverter with drawn filled tubing technology, where nitinol tubes with a platinum core are used to generate a braid of 48–64 wires.

In three cases, a Derivo2Heal was used as the FDS. The design of the Derivo2Heal is identical to the design of the Derivo peripher, but the Derivo2Heal has an antithrombogenic coating, due to which the extent and duration of the platelet function inhibition are reduced under certain clinical circumstances, for example, during acute hemorrhage.

In one case, a Pipeline Flex Shield was used. This is an FDS similar to the Derivo2Heal, with 48–64 wires composed of nitinol with a platinum core. This implant also has an antithrombogenic coating, allowing for reduced antiplatelet medication usage.

Depending on the diameter, a 0.021 in. microcatheter (Phenom 21, Medtronic, Dublin, Ireland), a 0.027 in. microcatheter (for example, Neuroslider 27, Acandis, Pforzheim, Germany or Phenom 27, Medtronic, Dublin, Ireland), or a 0.039 in. microcatheter (Neuroslider 39, Acandis, Pforzheim, Germany, or Phenom Plus, Medtronic, Dublin, Ireland) was used to implant the FDS in the respective artery.

### Patients and lesions

**Figure 1:**
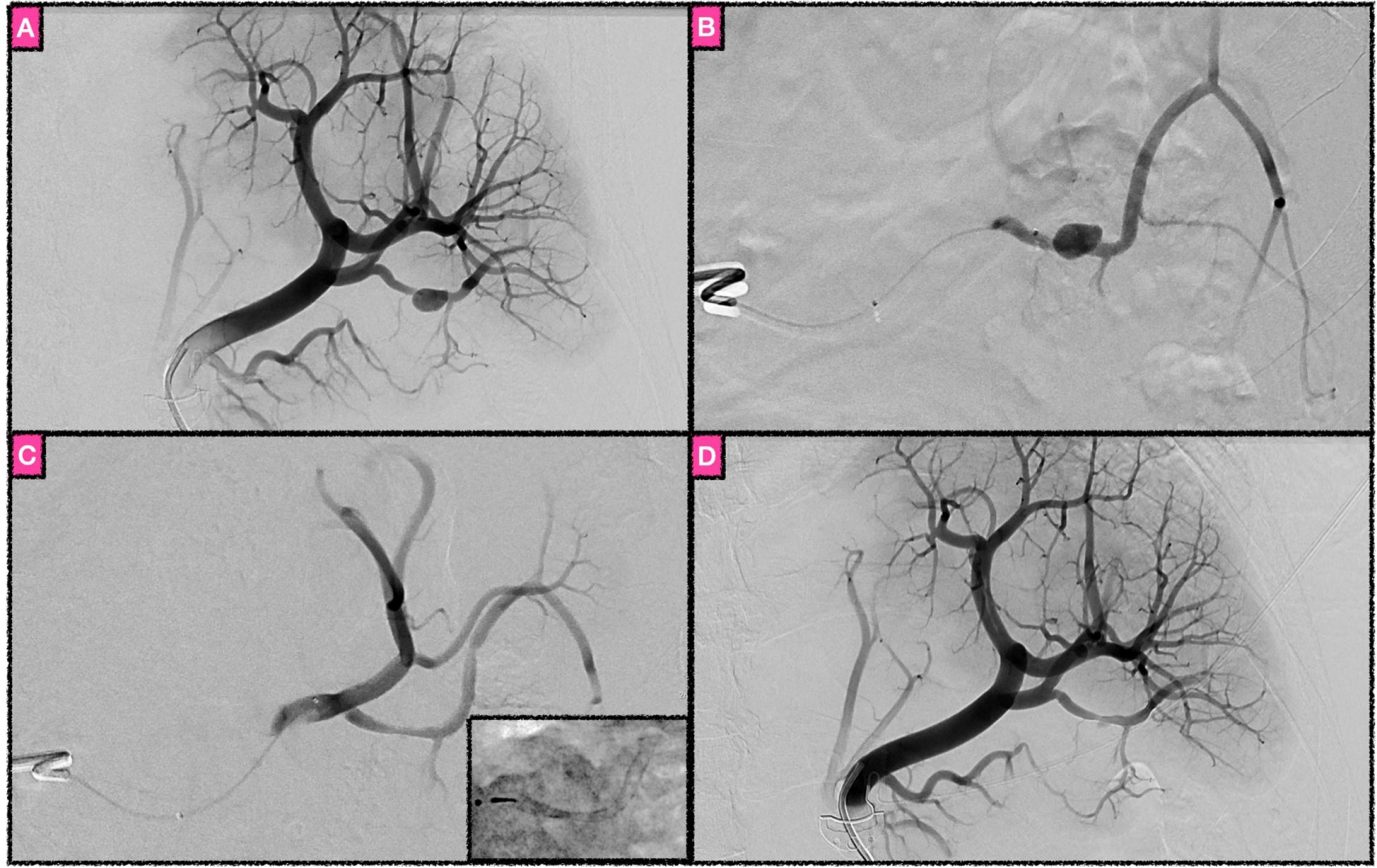
Flow diversion as treatment of a ruptured saccular aneurysm of an inferior polar branch of the splenic artery. **(A)** DSA run in the arterial phase revealing the aneurysm. **(B)** Magnified DSA image of the target vessel. The contrast agent injection was performed via a 0.021 in. microcatheter, superselectively opacifying the inferior polar artery in order to choose the most suitable FDS. **(C)** Target vessel after implantation of a Pipeline Vantage Flow Diverter via microcatheter. The FDS (shown in the fluoroscopic image in the right inferior corner) immediately triggered the occlusion of the aneurysm. Note that all three covered side branches remain patent, while the aneurysm is no longer opacified. **(D)** Final control run of the splenic artery, with a small neck remnant of the aneurysm, corresponding to O'Kelly–Marotta grade C3.

**Figure 2:**
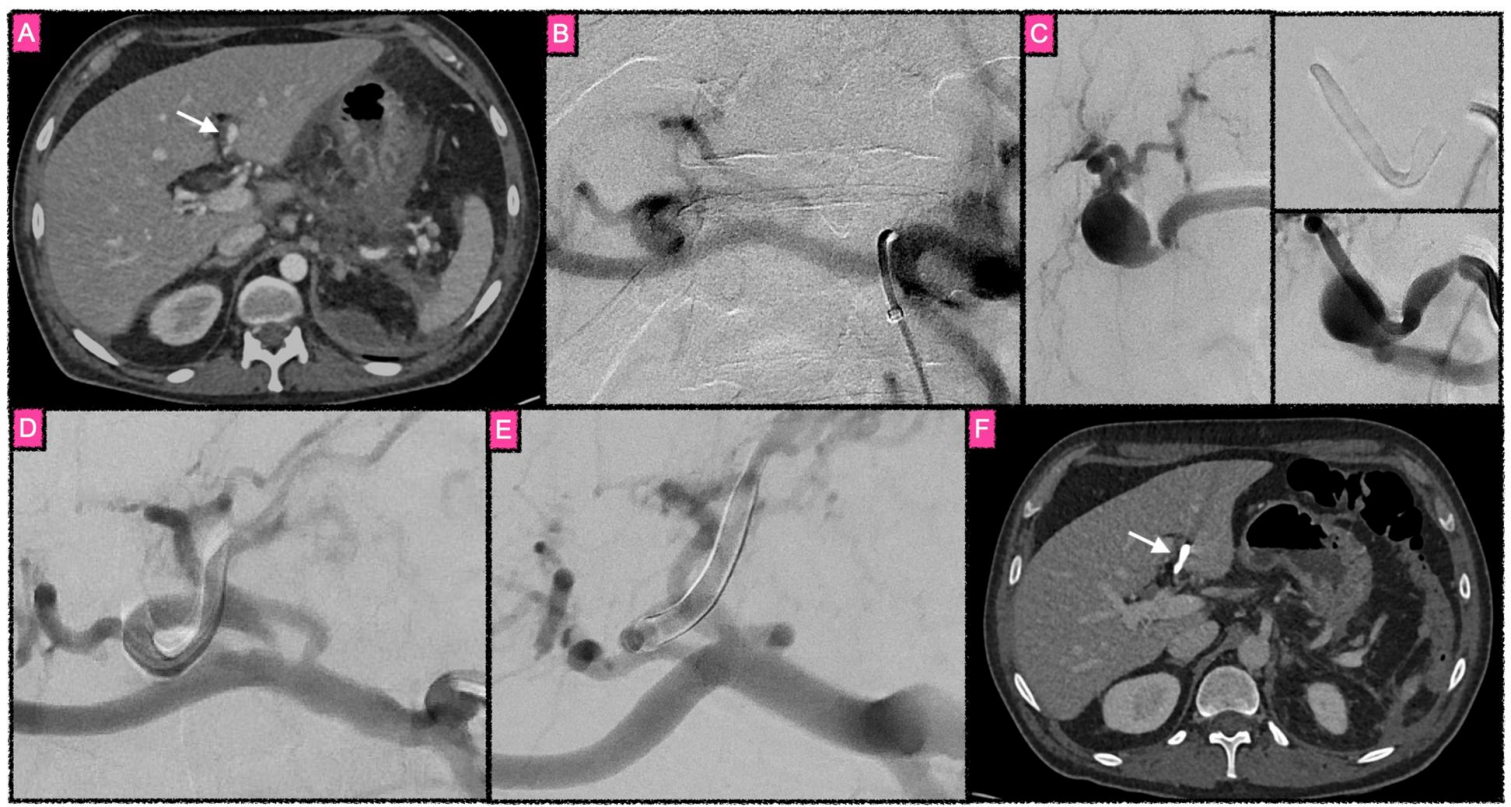
Treatment of an inflammatory aneurysm of the left hepatic artery. **(A)** Transversal computed tomography image in the early portal venous phase reveals a partially thrombosed aneurysm of the proximal left hepatic artery as a consequence of acute pancreatitis (white arrow). Follow-up ultrasound imaging (not shown) demonstrated rapid growth of the aneurysm within one week after the CT was performed, which was triggered by an episode of acute pain in the right upper quadrant of the patient's abdomen. Given the high risk for rupture and the anatomy at hand, endovascular treatment was selected. **(B)** Irregular aneurysm at the proximal left hepatic artery. **(C)** Course of FD treatment. The left side of **(C)** represents a microcatheter DSA run of the parent vessel prior to the implantation. There is significant vasospasm just proximal to the aneurysm, indicative of a previous rupture of the aneurysm. The right upper image in **(C)** shows the stent-in-stent construct within the parent vessel. A total of four FDS were implanted in an overlapping fashion in order to maximize the flow-diverting effect. **(D)** Early occlusion of the aneurysm after finalizing the multi-FDS implantation. The strong FD effect of the four implants in telescoping technique caused the almost immediate complete thrombosis of the aneurysm. **(E)** Follow-up DSA 3 months after treatment. The aneurysm is completely isolated from the circulation, the parent vessel remains patent. **(F)** Axial CT image 3 months after treatment, with the aneurysm thrombosed and shrunken (white arrow).

**Figure 3:**
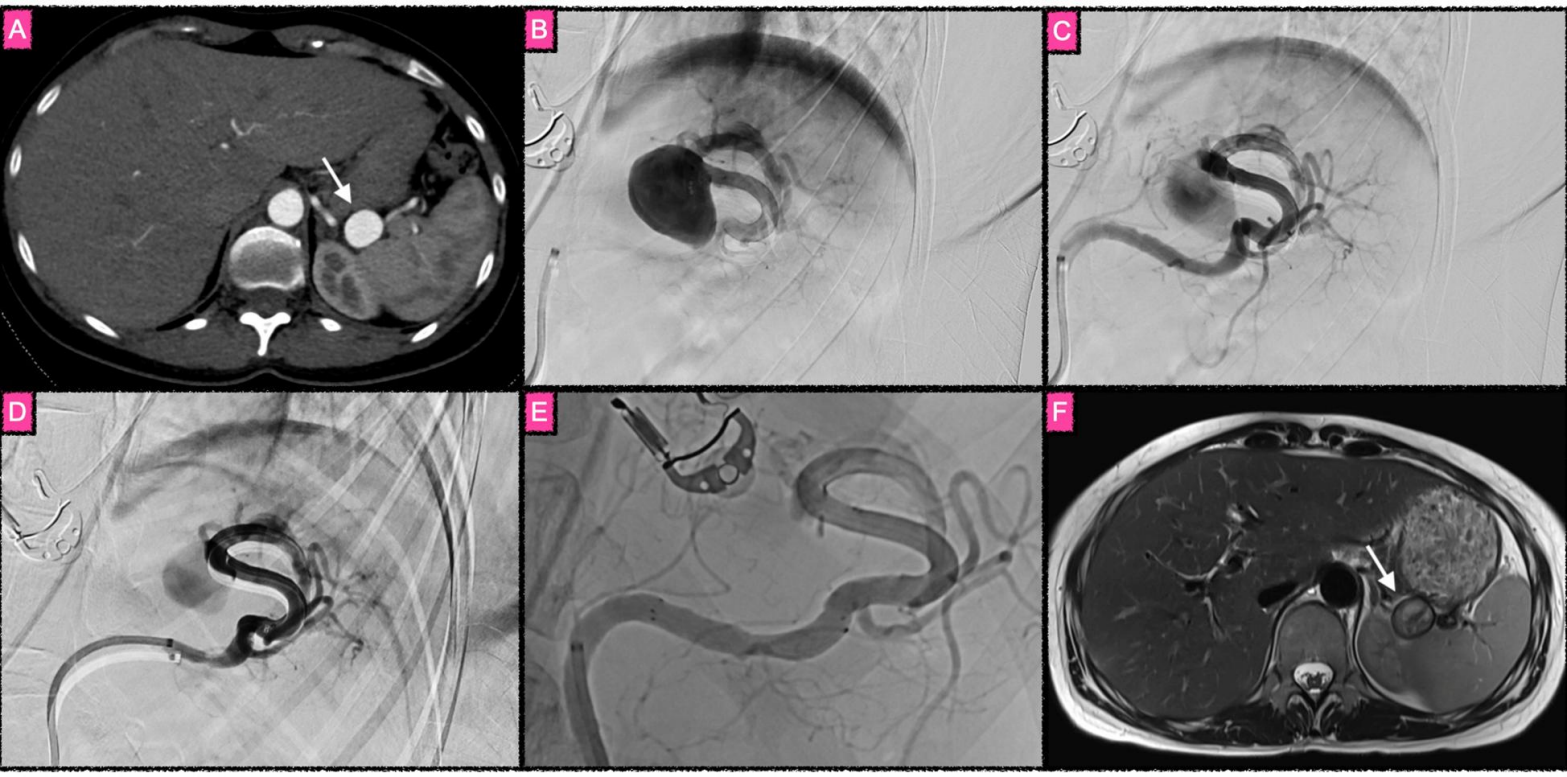
Treatment of a giant main stem aneurysm of the splenic artery with four overlapping flow diverter stents, equipped with antithrombogenic HEAL coating under prasugrel monotherapy. Given the large size of the aneurysm, the risk for delayed rupture after flow diversion–a rare but potentially fatal complication most likely occurring secondary to excessive thromboinflammation–was estimated to be comparatively high. Therefore, antiplatelet medication (prasugrel only) was reduced, based on the use of HEAL-coated FDS, which are significantly less thrombogenic and less proinflammatory. **(A)** Axial section of a computed tomography angiography in the arterial phase with the aneurysm (white arrow) in close proximity to the spleen. **(B)** Early phase of a superselective catheter angiography of the splenic artery after implantation of two FDS, with two adjacent FDS layers already covering the aneurysm neck. The DSA run reveals that there is still strong inflow into the aneurysm sac, whereas the splenic artery distal to the aneurysm is not as strongly opacified as the aneurysm itself. To further reduce aneurysm perfusion, two more FDS were implanted, so that a total of four layers of FDS covered the aneurysm neck. **(C)** DSA run of the splenic artery after the third FDS was implanted. **(D)** DSA run after the fourth FDS was implanted. In **(C)**, the opacification of the aneurysm was already significantly decreased compared to **(B)**; the reinforced hemodynamic effect of the fourth implant is visible in **(D)**. Catheter manipulations and subsequent vasospasm caused a strong fishmouth-like deformation of the proximal landing zone of the FDS construct, resulting in a significantly impaired perfusion of the splenic artery. Therefore, a self-expanding laser-cut stent (Acclino Flex Plus) was implanted into the stenosing proximal landing zone, which resolved the issue. **(E)** Unsubstracted DSA run of the splenic artery with the final stent construct in place. Approximately 10 min after implantation of the four overlapping FDS, clot had formed that almost completely occluded the aneurysm, corresponding to an O'Kelly–Marotta scale grade C3. Note the regular opacification of covered side branches of the splenic artery in the presence of the FDS telescoping construct. **(F)** Follow-up MRI (T2weighted axial image) 3 months after the procedure revealed organized thrombus completely filling the splenic artery aneurysm (white arrow), with the artery regularly perfused (flow void distal to the aneurysm), and no splenic infarction observable.

**Figure 4:**
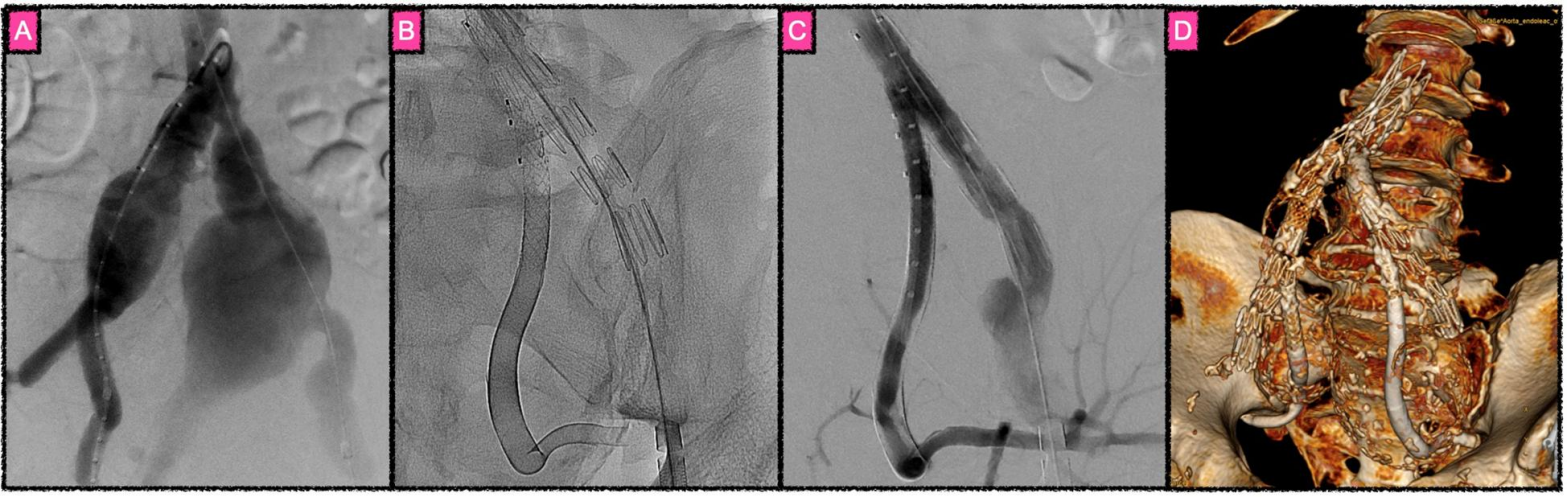
Complex reconstruction of a giant fusiform aneurysm involving the common iliac artery bifurcation and especially the external iliac artery, focusing on long-distance flow diversion for reconstruction of the external iliac artery. **(A)** Angiographic overview of the aortic bifurcation, both common, external and internal iliac arteries in right anterior oblique projection. At first, the left common iliac artery bifurcation was reconstructed performing T-stent grafting in order to generate a suitable proximal landing zone for the intended FDS construct. Then, the left internal iliac artery was antegradely microcatheterized until reaching the healthy vessel segment distal to the aneurysm. Subsequently, the first FDS was implanted in the distal healthy segment to create a stable anchoring point for the following FDS telescoping construct. Thereafter, five additional FDS were implanted in an overlapping manner from distal to proximal. Given the fusiform nature of the aneurysm, each FDS foreshortened maximally, as no wall attachment was achievable in the aneurysmatic vessel. The last, most proximal FDS was landed within the T-branch of the stent graft construct. The FDS were implanted with a minimum of 30% material overlap, so that sufficient stability of the telescoping construct was guaranteed. **(B)** Radiographic image of the FDS implants in the left-hand side internal iliac artery. A total of six FDS cover the entire aneurysm with a secure distal landing zone in the healthy segment and a secure proximal landing zone in the T-branch of the stent graft construct. **(C)** Subtracted DSA image after reconstruction. Besides the common and external iliac artery, only the lumen within the FDS construct in the internal iliac artery is opacified; the aneurysm outside the stents is already occluded by thrombus. Despite the high density of the braid mesh created by the overlapping FDS, covered side branches of the internal iliac artery remain sufficiently perfused in a timely normal fashion. **(D)** 3D reconstruction of a computed tomography performed 3 months after reconstructing the contralateral fusiform aneurysm in a similar manner.

**Figure 5:**
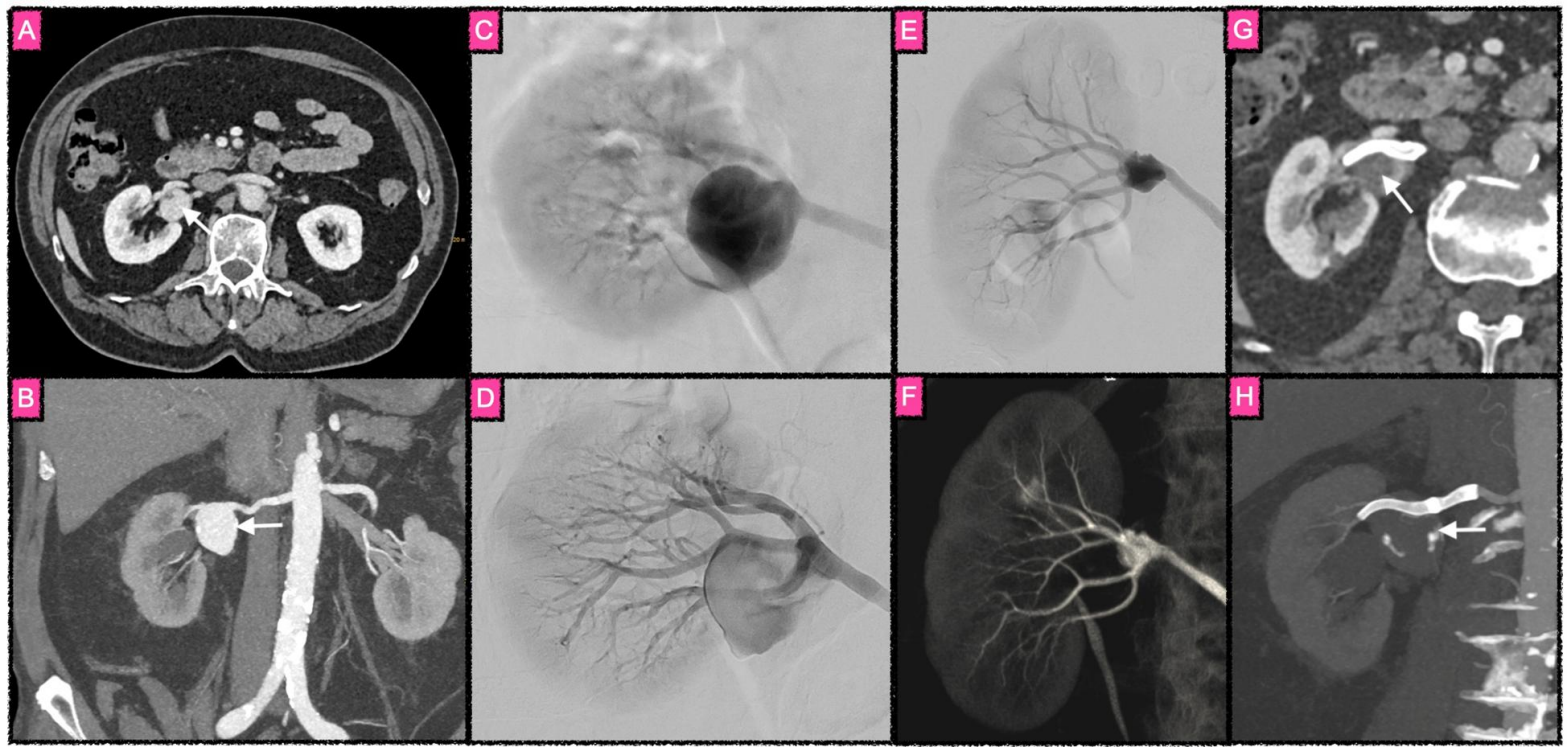
Course of FDS treatment of an aneurysm involving the right renal artery trifurcation. **(A)** and **(B)** Axial and coronal computed tomography angiographic images of the large renal artery aneurysm (white arrows) prior to treatment. **(C)** Superselective catheter angiogram of the right renal artery prior FDS implantation. The broad neck of the aneurysm is not clearly visualized in the images prior to treatment. **(D)** After implantation of two slightly overlapping FDS, with the distal landing zone in the superior branch of the renal artery trifurcation and the proximal landing zone in the renal artery main stem, the aneurysm is less opacified. The neck is clearly visible and contrast agent stagnation is depicted until the late parenchymal phase, corresponding to O'Kelly–Marotta scale grade A2. **(E)** Superselective control DSA 6 months after treatment with a reduced volume of the perfused aneurysm sac and contrast agent stagnation until the venous phase, corresponding to O'Kelly–Marotta scale grade B3. **(F)** Corresponding image of a 3D rotational angiography at the six months follow-up. **(G)** and **(H)** Axial and coronal computed tomography angiographic images of the aneurysm (white arrows) twelve months after treatment with a small residually perfused remnant close to the FDS construct, corresponding to O'Kelly–Marotta C3. Covered segmental arteries remain perfused, and there is no renal infarct visible.

A total of 36 patients, with 22 women with a mean age of 59.2 years (36–80 years), were treated with FD for aneurysms in the arterial territory of the spleen (*n* = 18), kidney (*n* = 7), liver (*n* = 8), superior mesenteric artery (*n* = 1), gastroduodenal artery (*n* = 1), external iliac artery (*n* = 1), and knee (*n* = 1). One patient had two aneurysms (splenic and mesenteric). Therefore, 36 patients with 37 aneurysms were analyzed. Table 1 summarizes the details of each treatment. Figures 1–5 provide examples of different flow diversion strategies for peripheral aneurysms. Figure 1 demonstrates the treatment of a small ruptured aneurysm of the inferior polar branch of the splenic artery. Figure 2 shows the treatment of an acutely ruptured aneurysm of the hepatic artery. Figure 3 depicts the treatment of a large incidental splenic artery main stem aneurysm. Figure 4 examplifies the complex endovascular approach for an iliac artery bifurcation aneurysm and Figure 5 shows the temporal course of flow diversion of a large right renal artery bifurcation aneurysm. A single FDS was implanted in 17 cases, two FDSs in 11 cases, three FDSs in three cases, and four, five, and six FDSs were implanted in one case each. In two patients, the diameter of the parent vessel segment measuring approximately 10 mm was too large for primary flow diversion; therefore, a larger braided stent was used to create sufficient landing zones prior to implantation of the FDS.

A total of 31 patients were treated with the Derivo peripher FDS, four patients with the Derivo2Heal FDS, one patient with both a Derivo peripher and a Derivo2Heal, and one patient with a Pipeline Vantage FDS. The choice of the implant depended on the availability of FDS in the respective center, the clinical circumstances, and the preference of the operator. The Derivo2Heal and the Pipeline Vantage were primarily chosen in order to reduce the antiplatelet medication earlier than usual, in case it was clinically required. Additional coiling was not performed in any of the cases. Further stents were required for technical success in six cases; the details are provided in the following.

### Imaging protocol

The interventional procedure and immediate postinterventional imaging required digital subtraction angiography in all cases. Supraselective angiographies of the parent vessels were performed prior to and after the interventional treatment. Fluoroscopic imaging was used for the deployment of the implants.

Depending on the standard of care in each vascular center, follow-up imaging was performed using digital subtraction angiography, computed tomography angiography, or magnetic resonance imaging angiography, allowing the assessment of the respective aneurysm and parent vessel.

In the case of follow-up studies employing computed tomography, an imaging study was performed, consisting of an unenhanced scan prior to contrast agent administration in order to assess the status of the dependent organ and the extent of aneurysmal thrombosis, followed by an arterial phase scan and a venous phase scan. In the case of follow-up studies employing magnetic resonance tomography, T2-weighted scans were performed to assess the status of the dependent organ, followed by an arterial phase and a venous phase MR angiogram after contrast agent administration.

As the angio suites, CT scanners, and MR scanners differed between the centers, detailed imaging protocols for each scanner were not included in our retrospective study.

### Antiplatelet medication

Dual antiplatelet therapy was administered to 32 patients with ASA used as the first drug and clopidogrel (*n* = 22), prasugrel (*n* = 10), or ticagrelor (*n* = 1) used as the second drug. Mono antiplatelet therapy was administered in three cases, one with prasugrel, one with ticagrelor (both patients had FDSs with anithrombogenic coating), and one with ASA as the antiplatelet in combination with edoxaban (cardiologic indication).

After 3 months, the second antiplatelet drug was stopped, and ASA 100 mg was continued as lifelong therapy.

One patient was treated with ticagrelor mono antiplatelet therapy under the circumstances of acute hemorrhage. The patient was loaded with ticagrelor (180 mg) 30 min prior to the procedure.

One further patient was treated with prasugrel mono antiplatelet therapy due to an extraordinarily large aneurysm with a higher risk of delayed rupture. Loading was performed 24 h prior to the procedure with 30 mg prasugrel. In both cases, sufficient platelet function inhibition was verified after loading using platelet function testing as reported previously (16).

In both cases of mono antiplatelet therapy, the medication was given for 3 months and then changed to lifelong ASA mono therapy.

### Technical success, procedural complications, complications during the follow-up period, and aneurysm occlusion

A technically successful FDS implantation was achieved in all patients. Two FDS treatments required the prior implantation of a larger braided stent in order to create suitable landing zones for the FDS. In both cases, in order to create sufficient and stable landing zones, the Accero Rex braided stent (10 mm × 60 mm and 10 mm × 30 mm, Acandis, Pforzheim, Germany) was placed first to enable implantation of the flow diverter stent, the first in the splenic artery and the second in the common hepatic artery.

In terms of technical complications, fishmouthing and stent instability at the proximal landing zone were the only relevant procedure-related complications and occurred in six cases, four of them during the procedure and two cases presenting at the first follow-up. In order to prevent subsequent occlusion of the parent artery, additional stentings were performed. In two cases, a self-expanding stent was used to reconstruct the proximal landing zone, which worked sufficiently, with details provided in Table 2. For this, the FDS-carrying segment was microcatheterized, and an Acclino Flex Plus (Acandis, Pforzheim, Germany) stent was implanted, with the center of the Acclino Flex covering the narrowed end of the crimped Derivo peripher. In the third case, an Accero Rex braided stent was implanted to optimize the landing zone at the end of the intervention. In the fourth case, a Formula balloon-mounted stent (Cook Medical, Bloomington, Indiana, USA) was used to revise the proximal fishmouthing successfully. The last case of proximal fishmouthing manifested in the early postinterventional period and was diagnosed at the first follow-up. In this case, a Formula balloon-mounted stent (Cook Medical, Bloomington, Indiana, USA) was implanted to optimize the proximal landing zone. Thus, fishmouthing was managed successfully in all cases.

In a singular case, the FDS construct became partially dislocated into the aneurysm due to the lack of a sufficient landing zone in the parent vessel. In this case, the FDS construct was stabilized proximally with a Formula balloon-mounted stent (Cook Medical, Bloomington, Indiana, USA), resulting in satisfying positioning of the implants.

In two cases, occlusion of the FDS occurred, which remained asymptomatic at all times.

In one case, the occlusion of the FDS manifested during the intervention and was related to clopidogrel resistance. The patient was treated in an elective setting for aneurysms of the splenic artery and a side branch of the superior mesenteric artery. Control injections at the end of the procedure showed thrombotic occlusions of the distal splenic artery (with good collateralization via splenic artery branches) and the occlusion of a well-collateralized branch of the superior mesenteric artery. Multiplate platelet function testing was performed and revealed high platelet reactivity in the ADP test, despite sufficient clopidogrel having been administered; sufficient platelet function inhibition by ASA was verified. A bolus of Integrilin was given to manage the high platelet reactivity due to clopidogrel resistance, and aspiration thrombectomy was also performed. However, recanalization could not be achieved. Antiplatelet medication with clopidogrel was stopped, and ASA was continued. Control imaging (CT with CTA) was performed the next day and revealed partial splenic infarcts, which remained asymptomatic. There was no intestinal ischemia. The patient remained asymptomatic and left the hospital three days after the procedure. The condition of the patient at 3 and 12 months following the procedure remained unchanged.

In the second case, a patient was treated for a large bifurcation aneurysm of the right-hand side renal artery with two FDSs. The procedure was technically uneventful, and the control CT the day after the intervention showed a well-perfused renal artery after stenting. However, due to intraperitoneal bleeding of unclear cause three months after the intervention, which was treated at another hospital, both antiplatelet drugs were stopped, which resulted in a thrombotic occlusion of the stents. The patient suffered from a partial infarction of the right kidney. Function of the left kidney and the remaining parenchyma of the right kidney remained sufficient, and the patient recovered completely from the complication.

**Figure 6:**
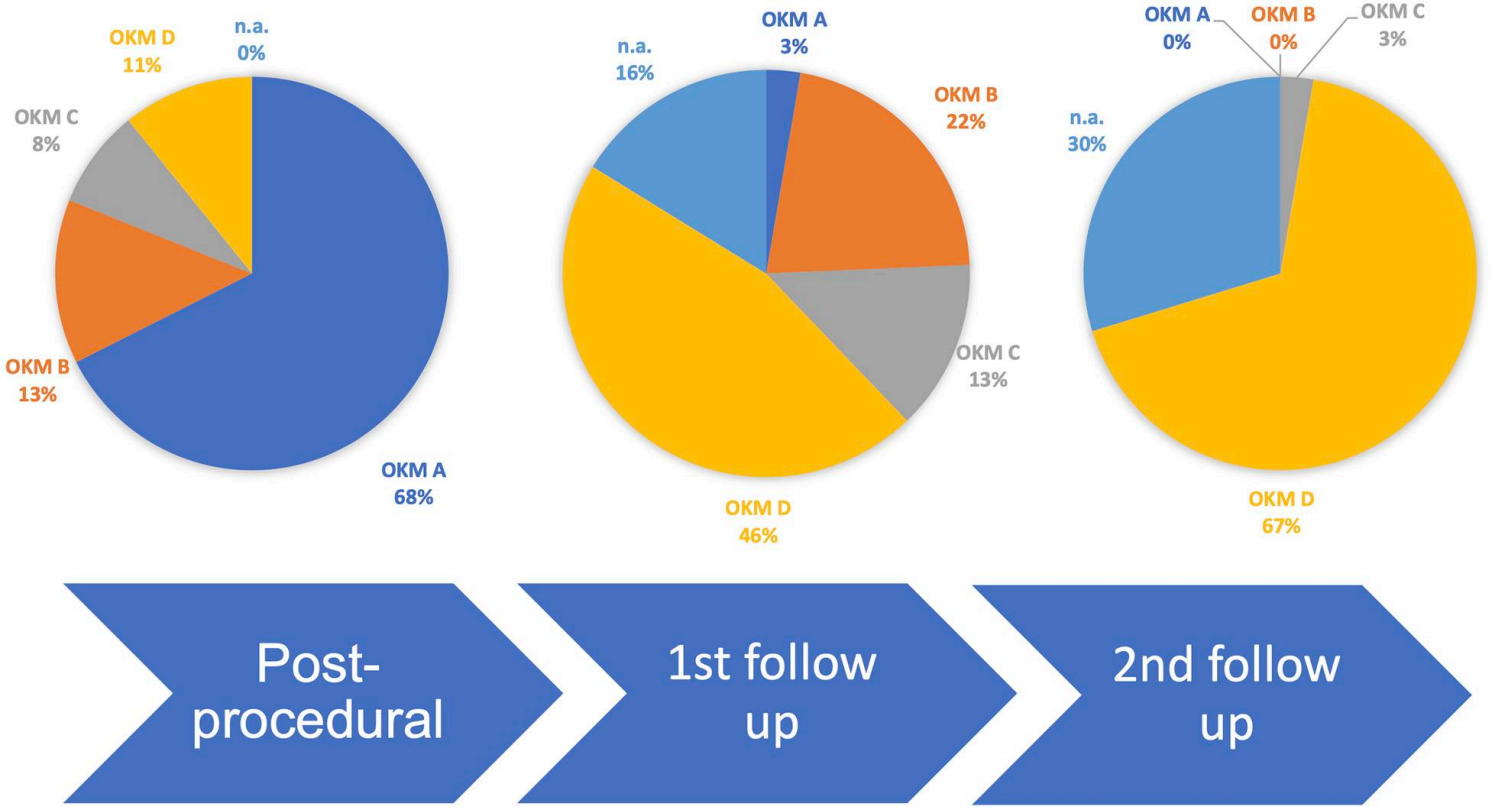
Flowchart of follow-up studies and corresponding occlusion rates.

**Figure 7:**
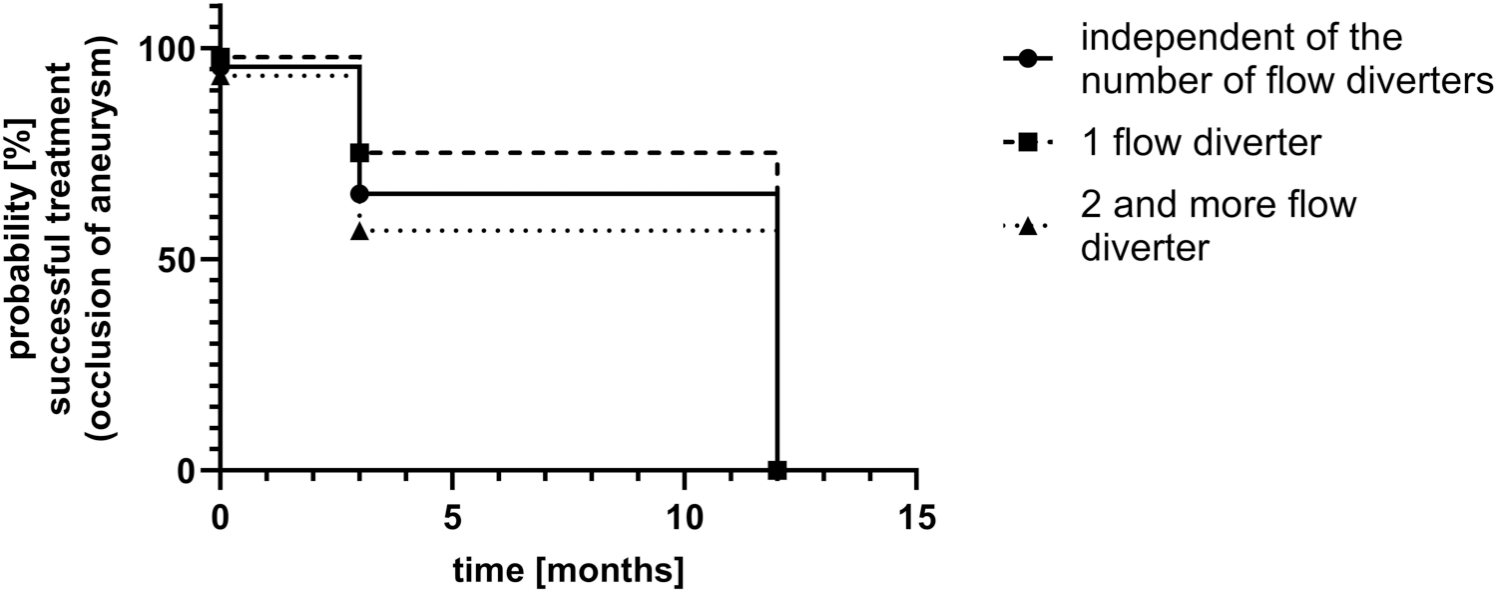
Kaplan–Meier graph of aneurysm occlusion over time.

A total of four aneurysms were occluded immediately at the end of the procedure. In 33 aneurysms, the perfusion was reduced significantly, but to a varying extent. In the first follow-up study, 31 aneurysms were assessable. At the first follow-up, scheduled after 3 months, 17 aneurysms were occluded, 14 aneurysms showed a reduced perfusion, and six had not yet been evaluated. At the 12-month follow-up, 26 lesions were assessed. A total of 27 aneurysms were occluded after 12 months, 17 of which had already been occluded at the 3-month study. Two of these did not undergo a follow-up at 12 months. One aneurysm showed a small neck remnant. Overall, 11 aneurysms did not undergo a second follow-up study. Table 3 provides an implant and strategy based comparison of the cases. Figure 6 shows a comprehensive timeline of aneurysm occlusion in the follow-up flowchart. Figure 7 provides a Kaplan–Meier graph depicting overall time to occlusion, time to occlusion in case only one flow diverter was used, and time to occlusion in case two or more flow diverters were implanted via a telescoping technique.

**Table 3:** Device-based, comparative summary of the study results. In the case of two or more overlapping flow diverter stents (telescoping technique), a minimum device overlap of 30% was performed, except for the cases of treatments of acutely ruptured aneurysms.

Device	Derivo peripher	Derivo2Heal	Derivo peripher + Derivo2Heal	Pipeline vantage
Total number of patients treated	31	4	1	1
Number of patients with one device	16	1	0	1
Number of patients with two or more overlapping devices	15 (Two implants: 11) (Three implants: 3) (Six implants: 1)	3 (Three implants: 1) (Four implants: 1) (Five implants: 1)	1 (One uncoated and one coated implant)	0
Lesion types	*Splenic artery: n* = 17 (all saccular unruptured) *Renal artery: n* = 6 (all saccular unruptured) *Hepatic artery:* *n* = 7 (2 ruptured, 1 fusiform unruptured, 4 saccular unruptured aneurysms) *Sup. mesenteric artery:* *n* = 1 (saccular unruptured aneurysm) *Ext. iliac. artery:* *n* = 1 (fusiform unruptured aneurysm)	*Splenic artery: n* = 1 (saccular unruptured, rapidly growing aneurysm) *Renal artery:* *n* = 1 (saccular unruptured aneurysm, patient suffering also from multiple cerebral aneurysms related to fibromuscular dysplasia) *Hepatic artery:* *n* = 2 (both saccular ruptured aneurysms). Derivo2Heal was used to allow early de-escalation of antiplatelet therapy due to hemorrhage	*Inf. genicular artery: n* = 1 Flow-related aneurysm distal to a peripheral AVM Derivo2Heal was used to decrease the risk of occlusion of the covered branch supplying the fibular nerve	*Splenic artery:* *n* = 1 (ruptured saccular aneurysm) Pipeline vantage was used to allow early de-escalation of antiplatelet therapy due to hemorrhage
Technical success	100%	100%	100%	100%
Further stents required	*n* = 5 cases 2×Accero Rex 2×Cook Formula 1×Acclino Flex Plus	*n* = 1 case 1×Acclino Flex Plus	No	No
Complications	*n* = 2, both related to stent occlusion due to insufficient antiplatelet therapy	None	None	None
Occlusion rates post procedurally	OKM A: *n* = 23 OKM B: *n* = 3 OKM C: *n* = 1 OKM D: *n* = 4 NA: *n* = 0	OKM A: *n* = 1 OKM B: *n* = 2 OKM C: *n* = 1 OKM D: *n* = 0 NA: *n* = 0	OKM A: *n* = 1 OKM B: *n* = 0 OKM C: *n* = 0 OKM D: *n* = 0 NA: *n* = 0	OKM A: *n* = 0 OKM B: *n* = 0 OKM C: *n* = 1 OKM D: *n* = 0 NA: *n* = 0
Occlusion rates first followup	OKM A: *n* = 1 OKM B: *n* = 8 OKM C: *n* = 4 OKM D: *n* = 12 NA: *n* = 6	OKM A: *n* = 0 OKM B: *n* = 0 OKM C: *n* = 1 OKM D: *n* = 3 NA: *n* = 0	OKM A: *n* = 0 OKM B: *n* = 0 OKM C: *n* = 0 OKM D: *n* = 1 NA: *n* = 0	OKM A: *n* = 0 OKM B: *n* = 0 OKM C: *n* = 0 OKM D: *n* = 1 NA: *n* = 0
Occlusion rates second followup	OKM A: *n* = 0 OKM B: *n* = 0 OKM C: *n* = 1 OKM D: *n* = 19 NA: *n* = 11	OKM A: *n* = 0 OKM B: *n* = 0 OKM C: *n* = 0 OKM D: *n* = 4 NA: *n* = 0	OKM A: *n* = 0 OKM B: *n* = 0 OKM C: *n* = 0 OKM D: *n* = 1 NA: *n* = 0	OKM A: *n* = 0 OKM B: *n* = 0 OKM C: *n* = 0 OKM D: *n* = 1 NA: *n* = 0

In those patients, a higher overlap (50%–100%) was achieved in order to increase the flow-diverting effect as much as possible, so that aneurysm perfusion could be reduced maximally with early aneurysm occlusion being more likely. An ÒKelly–Marotta occlusion class A indicates a completely perfused aneurysm, class B indicates a significant decrease in size with more than a neck remnant being visible, class C corresponds to a neck remnant, and D indicates a completely occluded aneurysm.

## Discussion

FDSs were first designed to treat uncoilable or failed aneurysms of the internal carotid artery from the petrous to the clinoid segment (17). With convincing evidence relating to the success of the novel therapeutic approach, indications grew rapidly and FD was employed to treat aneurysms in the posterior circulation, bifurcation aneurysms of the circle of Willis, dissecting aneurysms, and very distal aneurysms arising from small-caliber cerebral arteries (18–22). Furthermore, the results of the first studies have demonstrated that the flow-redirecting and vascular remodeling effects of the FDS can also be used to indirectly treat aneurysms, which cannot directly be covered with an FDS because of anatomical circumstances (23). This technique—indirect or remote flow diversion—employs FDS in a main artery for the treatment of aneurysms arising distally from small-caliber parent arteries and has shown satisfactory results thus far.

The treatment of splenic, hepatic, renal, and visceral or peripheral arterial aneurysms can be technically as challenging as the treatment of cerebral arterial aneurysms for similar reasons (24). Visceral aneurysms and pseudoaneurysms are rare yet potentially fatal lesions with mortality rates between 25% and 100% in case of rupture (25). Therefore, depending on localization, size, and further risk factors, treatment is recommended if the risk criteria are met (26). Accompanying the rapid development of endovascular tools, endovascular treatment has become the preferred approach compared to open surgery over the past few decades as available evidence suggests that the rate of perioperative complications is higher and hospital stays are prolonged in patients treated via open surgery, while aneurysm occlusion rates are similar (27). However, are a variety of endovascular techniques are currently being employed, including reconstructive techniques such as stent grafting and stent- and ballon-assisted coiling, as well as deconstructive techniques employing liquid embolic agents, vascular plugs, and coils for parent artery occlusion or segmental vessel sacrifice (24). In general, reconstruction of the parent artery is always preferable over sacrifice, as the occlusion of a main artery or a segmental artery supplying an organ carries a substantial risk of infarction and subsequently the loss of essential organ function. In the case of splenic artery aneurysms, for example, vessel embolization may not only cause splenic infarction but can also result in ischemic pancreatitis, ultimately requiring partial pancreatectomy and splenectomy (28–30). A number of studies have linked splenic function not only to the prevention of severe infections and sepsis but also to deep vein thrombosis, pulmonary embolism, and cancer (31). It is of utmost importance to prevent ischemia in other affected abdominal organs, like the liver and the kidneys, by preserving arterial flow in context of aneurysm repair.

Unfortunately, traditionally established reconstructive techniques, of which the most important is stent grafting, are oftentimes not applicable as the delivery systems are stiff and require large bore vascular access, which is not always compatible with the substantial tortuosity of the visceral parent vessels. Furthermore, insufficient stent-wall apposition and endoleaks in tortuous vascular anatomy are well-known obstacles for stent grafts (10, 32).

Microcatheters required for FDS implantation have much smaller diameters and offer much greater flexibility compared to stent graft systems. Therefore, lesions not amendable for conventional reconstructive endovascular aneurysm repair have become treatable with FDSs. Our study is among the first providing evidence relating to the safety and feasibility of FD treatment for visceral and peripheral aneurysms. First of all, all aneurysms in our patient cohort were successfully treatable with FD—each parent vessel was eventually navigable with the microcatheters required for FDS implantation; even the substantial tortuosity of the splenic artery and the left hepatic artery, as exemplarily demonstrated in Figures 2, 3, did not result in insurmountable technical obstacles.

Second, considering the results of the available follow-up studies up to 12 months after the procedure, 82% of the treated aneurysms were completely occluded after approximately three months. This largely corresponds to the occlusion rates published in the context of intracranial aneurysm treatment with FDS, where more than 75% of the aneurysms are occluded within the first year after treatment (5).

Importantly, no side branch occlusion occurred, and no ischemic or hemorrhagic complications manifested in the patients who were treated with uninterrupted suitable antiplatelet therapy. This underscores the general safety and efficacy of FD as an alternative treatment modality for visceral and peripheral aneurysms.

However, two patients experienced occlusions of their (uncoated) flow diverter implants with partial end-organ infarctions. In the first case, the occlusion was related to an acute abdominal hemorrhage of undetermined origin during the early phase of dual antiplatelet therapy, which was terminated because of the bleeding, resulting in occlusion of the renal artery FDS construct and infarction of approximately 50% of the dependent kidney parenchyma. In the second case, occlusion of the FDS in the splenic artery and in a branch of the superior mesenteric artery occurred during the intervention and was related to clopidogrel resistance, which was not detected previously. The patient developed significant splenic infarctions; no intestinal ischemia occurred due to sufficient collateralization. Both cases—despite the eventual full recovery of the patients—substantiate the importance of continuous antiplatelet therapy and preinterventional platelet function testing (16, 33–35) for stent-based aneurysm repair, not only in cerebral circulation but also in the context of visceral and peripheral aneurysms. Considering the currently available evidence regarding the high rate of clopidogrel resistance and the substantial thrombogenicity of FDSs without antithrombogenic surface modification (36–38), precautions should be taken to further improve the safety profile of flow diverter treatments. To achieve this, platelet function testing should be performed prior to each intervention in a standardized manner, P2Y12 inhibition should be approached with drugs less prone to high on-treatment platelet reactivity, such as ticagrelor and prasugrel, and only implants with proven antithrombogenic surface modification should be used under these circumstances (16, 38–40).

Recent studies investigating conventional endovascular treatments of visceral and peripheral aneurysms (coiling, stent grafting, liquid embolic agents, etc.) have demonstrated low complication rates of between 5.8% and 12.5% and high technical success rates of between 93.3% and 94.5% (25–27). In detail, the reported complications included both hemorrhagic and ischemic events; the ischemic complications were primarily related to material issues, while the hemorrhagic complications mostly involved secondary bleedings. In our study, no hemorrhagic complication occurred—despite the use of significant antiplatelet therapy. However, 5.4% of patients (two patients) experienced an ischemic complication. Both were related to insufficient inhibition of platelet function in combination with uncoated flow diverter stents. As compared to the above-mentioned studies, our complication rates are within currently acceptable limits. However, the use of implants with antithrombogenic coating in those cases might have had the potential to prevent both stent occlusions, especially when considering the absence of ischemic complications or stent occlusions in patients treated with coated flow diverters under mono antiplatelet therapy.

In terms of the technical success rate, our study has demonstrated the absence of technical failure in all cases. This indicates that flow diversion provides a valuable therapeutic option in those cases where other techniques, such as coiling and stent grafting, although on rare occasions, fail (25–27). Nevertheless, flow diversion should not be considered the first therapeutic option in those patients with aneurysms who are well suited for the conventional approaches. For example, patients suffering from side-wall aneurysms or pseudoaneurysms, which do not involve an arterial bifurcation, or patients who require anticoagulation for a preexisting condition would certainly benefit from the use of conventional techniques.

Our study suffers from a number of limitations. First, despite being a multicenter observational study, the number of patients is limited. Its retrospective nature, with a comparatively short follow-up period and incomplete follow-up studies in some cases, does not allow us to draw general conclusions on a larger scale. Second, there is substantial variability regarding the antiplatelet medication, a lack of standardized platelet function testing, heterogeneity regarding the use of implants with and without antithrombogenic surface modification, and the modality of follow-up imaging. In particular, the differing antiplatelet management processes of the centers may have influenced the outcomes substantially. First, the different P2Y12 antagonists have very distinct pharmacokinetic profiles. Second, prasugrel and ticagrelor are much more potent platelet inhibitors than clopidogrel. Thus, the use of those drugs may have prolonged the process of aneurysm occlusion compared to clopidogrel, as occlusion is directly linked to aneurysmal thrombosis. Third, high on-treatment platelet function is a well-known phenomenon related to clopidogrel usage, which in our study was responsible for the only complications in our patient collective.

Therefore, prospective randomized trials addressing the following questions are warranted: (1) Which antiplatelet therapy is optimal for the flow diverter treatment of visceral and peripheral aneurysms? (2) What is the required duration of antiplatelet therapy in this context? (3) Does the use of antithrombotically modified implants increase the safety of aneurysm treatments compared to uncoated implants? What is the long-term outcome, in particular the fate of the treated vessel and the dependent organ?

## Conclusion

Our multicenter observational study offers limited but valuable insight into the safety and feasibility of FD as a treatment modality for visceral and peripheral aneurysms in the case of anatomically challenging parent vessels and bifurcation aneurysms. Flow diversion for the treatment of visceral and peripheral aneurysms will significantly benefit from standardized therapeutic antiplatelet management.

## Data Availability

The original contributions presented in the study are included in the article/Supplementary Material, further inquiries can be directed to the corresponding authors.
